# Comparative Metabolomics and Proteomics Reveal *Vibrio parahaemolyticus* Targets Hypoxia-Related Signaling Pathways of *Takifugu obscurus*

**DOI:** 10.3389/fimmu.2021.825358

**Published:** 2022-01-13

**Authors:** Jiachang Xu, Xue Yu, Hangyu Ye, Songze Gao, Niuniu Deng, Yuyou Lu, Haoran Lin, Yong Zhang, Danqi Lu

**Affiliations:** ^1^ State Key Laboratory of Biocontrol and School of Life Sciences, Southern Marine Science and Engineering Guangdong Laboratory (Zhuhai), Guangdong Provincial Key Laboratory for Aquatic Economic Animals and Guangdong Provincial Engineering Technology Research Center for Healthy Breeding of Important Economic Fish, Sun Yat-Sen University, Guangzhou, China; ^2^ Laboratory for Marine Fisheries Science and Food Production Processes, Qingdao National Laboratory for Marine Science and Technology, Qingdao, China; ^3^ College of Ocean, Hainan University, Haikou, China

**Keywords:** fibrosis, cell proliferation, LRG, TGF-β1, HIF-1α, EGF

## Abstract

Coronavirus disease 2019 (COVID-19) raises the issue of how hypoxia destroys normal physiological function and host immunity against pathogens. However, there are few or no comprehensive omics studies on this effect. From an evolutionary perspective, animals living in complex and changeable marine environments might develop signaling pathways to address bacterial threats under hypoxia. In this study, the ancient genomic model animal *Takifugu obscurus* and widespread *Vibrio parahaemolyticus* were utilized to study the effect. *T*. *obscurus* was challenged by *V*. *parahaemolyticus* or (and) exposed to hypoxia. The effects of hypoxia and infection were identified, and a theoretical model of the host critical signaling pathway in response to hypoxia and infection was defined by methods of comparative metabolomics and proteomics on the entire liver. The changing trends of some differential metabolites and proteins under hypoxia, infection or double stressors were consistent. The model includes transforming growth factor-β1 (TGF-β1), hypoxia-inducible factor-1α (HIF-1α), and epidermal growth factor (EGF) signaling pathways, and the consistent changing trends indicated that the host liver tended toward cell proliferation. Hypoxia and infection caused tissue damage and fibrosis in the portal area of the liver, which may be related to TGF-β1 signal transduction. We propose that LRG (leucine-rich alpha-2-glycoprotein) is widely involved in the transition of the TGF-β1/Smad signaling pathway in response to hypoxia and pathogenic infection in vertebrates as a conserved molecule.

## Introduction

Severe acute respiratory syndrome coronavirus 2 (SARS-CoV-2) causes coronavirus disease 2019 (COVID-19). As of October 22, 2021, more than 242.3 million SARS-CoV-2 infections and 4.9 million COVID-19-related deaths have been documented (1, 2). A previous proteomic study of COVID-19 autopsies indicated dysregulation of key factors involved in hypoxia, angiogenesis, blood coagulation, and fibrosis in multiple organs from COVID-19 patients, and these factors contributed to typical histopathological features of COVID-19, such as microthrombi, proliferation of fibroblasts/myofibroblasts and fibrosis in alveolar septa, and intussusceptive angiogenesis (3, 4). COVID-19 raises the issue of how hypoxia destroys the normal physiological function and immunity of the human body and therefore promotes the invasion of SARS-CoV-2 (5, 6).

Physiological hypoxia plays a role in shaping innate and adaptive immunity and maintaining physiological homeostasis, while pathological hypoxia drives tissue dysfunction and disease development through immune cell dysregulation (7). A prominent instance of pathological hypoxia is inflammation. Usually, inflammation demands the recruitment of a large number of myeloid cells, and the severe metabolic burden and huge demand for energy for the transport of cells contribute to an increase in oxygen consumption (8–10). Therefore, host cells have developed strategies to adapt to the antibacterial response under hypoxia. For instance, neutrophils, which play a major role in countering infection, principally utilize glycolysis as a way to obtain energy (11), and an oxygen concentration as low as 4.5% does not significantly affect their respiratory burst activity (12); therefore, neutrophils recognize and phagocytize pathogens under hypoxia (13).

From an evolutionary perspective, animals living in complex and changeable marine environments might develop signaling pathways to address bacterial threats under hypoxia (14). In this study, *Takifugu obscurus* (an ideal model to study the vertebrate genome) and *Vibrio parahaemolyticus* (a common opportunistic pathogen in the temperate zone and the tropics) were utilized to study the effects of hypoxia on immunity against pathogens (15–17). The metabolomic and proteomic characteristics of the entire liver of *T*. *obscurus* exposed to hypoxia and/or infection with *V*. *parahaemolyticus* were comparatively analyzed, and a theoretical model of the critical signaling pathway in response to hypoxia and infection was defined. We propose that LRG (leucine-rich alpha-2-glycoprotein) is widely involved in the transforming growth factor-β1 (TGF-β1) signaling pathway in response to hypoxia and pathogenic infection in vertebrates as a conserved molecule.

## Results

### Hypoxia-Accelerated Deaths Caused by Infection of *V*. *parahaemolyticus* and Fibrosis Caused by the Two Stressors

**Figure 1:**
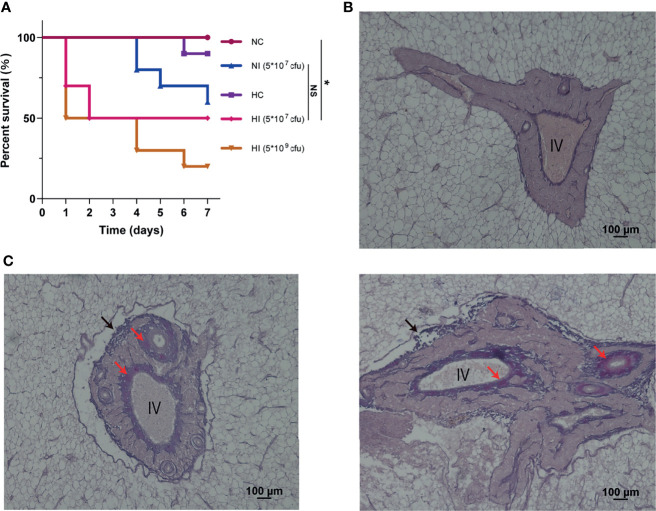
Survival curve and pathological observation of portal area. **(A)** The survival curve was measured in the independent preliminary experiment. For treatments of hypoxia and infection, *T*. *obscurus* were exposed to hypoxia for 12 h and then infected by *V*. *parahaemolyticus*. Finally, the survival rate of *T*. *obscurus* was monitored for 7 d. *n* = 10 biologically independent animals per group. *P* values were determined using two-sided log-rank (Mantel-Cox) tests. NS, not significant. *P value < 0.05. **(B)** Representative image from at least three biological replicates of the NC group. IV, interlobular vein. **(C)** Representative images of different stages of fibrosis from at least three biological replicates of the HI group. Black arrows, damaged walls; Red arrows, fibers.

We established and observed a model of *T*. *obscurus* under hypoxia and infection with *V*. *parahaemolyticus* for 7 days, and then the mortality rate was counted (**Figure 1A**). Deaths first occurred on Day 4 in the normoxic infection (NI) (5 * 10^7^ colony-forming unit, cfu) group, and the final survival rate after 7 days was 60%. Deaths occurred on Day 6 in the hypoxic control (HC) group, and the final survival rate was 90%. The number of deaths in the hypoxic infection (HI) (5 * 10^7^ cfu) group increased from the first day, and the final survival rate was 50%. The final survival rate in the HI (5 * 10^9^ cfu) group was 20%, which meant that the bacterial load affected the final mortality. Therefore, although hypoxia had no significant effect on the number of deaths caused by *V*. *parahaemolyticus* infection, it advanced the occurrence of deaths (**Figure 1A**). Pathological observation of the liver indicated that tissue damage and fibrosis occurred in the walls of interlobular veins, arteries or bile ducts in the portal area (**Figures 1B, C**). Deaths principally occurred on the first day (from 12 to 24 h); therefore, we anticipated that the host response in the early stage of infection would largely determine the final survival. Accordingly, metabolomic and proteomic analyses of the entire liver were performed at 12 h.

### Screening and Characterization of Differential Metabolites

**Figure 2:**
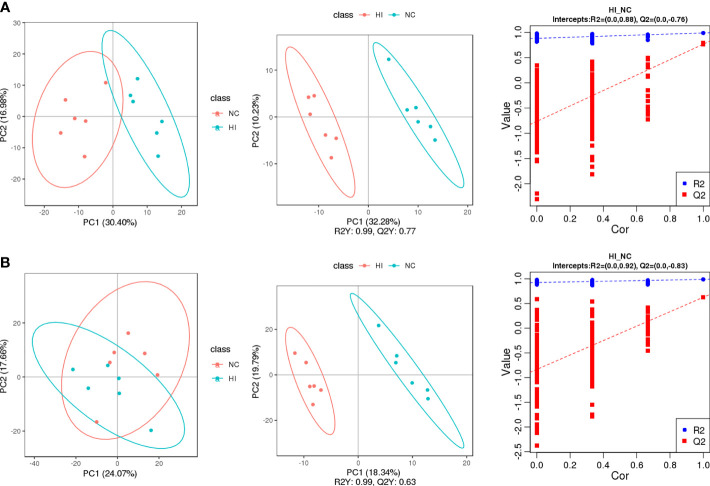
The screening of differential metabolites of the NC and HI groups was carried out for quality control and comparisons of differential metabolic characteristics. PC1 and PC2 represent the scores of the first and second principal components, respectively. **(A)** PCA, PLS-DA and function based on the sevenfold cross-validation method of the NC and HI groups in negative ion scanning mode. **(B)** PCA, PLS-DA and function based on the sevenfold cross-validation method of the NC and HI groups in positive ion scanning mode.

Principal component analysis (PCA) compared the differences in metabolic characteristics between the normoxic control (NC) and HI groups and the degree of variation between parallel samples in the group. Partial least squares discriminant analysis (PLS-DA) established a model of the relationship between metabolite production and sample categories, and variable importance in the projection (VIP) was obtained to assist in the screening of differential metabolites (**Figure 2**). The model demonstrated good explanatory and predictive ability (Neg: R^2^Y = 0.99, Q^2^Y = 0.77. Pos: R^2^Y = 0.99, Q^2^Y = 0.63). The method described before was used to indicate that overfitting had not occurred (R^2^ > Q^2^, y-intercept of Q^2^ < 0) (18). As expected, minor differences in metabolic characteristics of hypoxia or infection were obtained (**Figure S1**).

**Figure 3:**
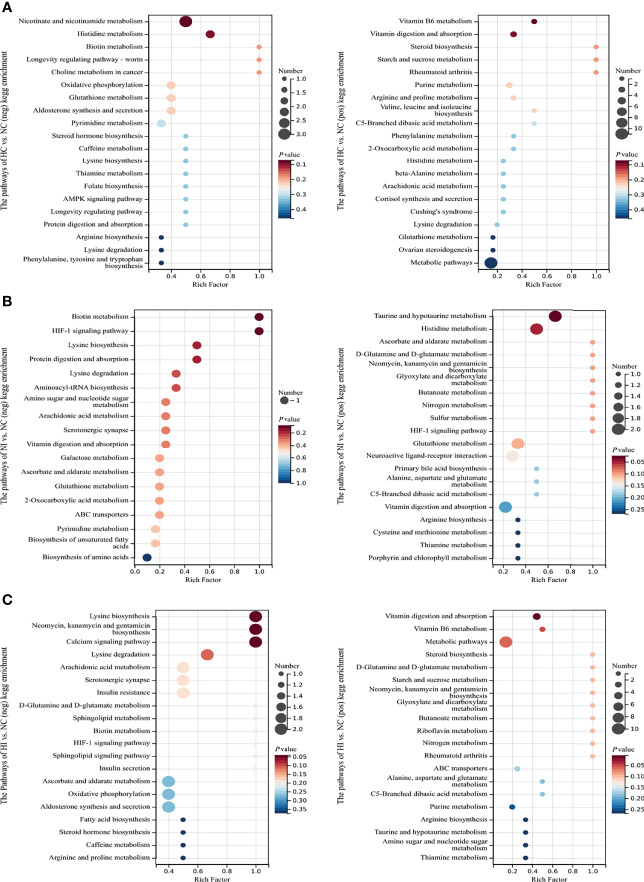
The enriched top 18 or 20 KEGG pathways sorted by *P* value based on the differential metabolites of the three compared groups. The size of the point indicates the number of enriched metabolites. Rich Factor, the ratio of *x* to *M*. **(A)** The enriched KEGG pathways of NC versus HC. **(B)** The enriched KEGG pathways of NC versus NI. **(C)** The enriched KEGG pathways of NC versus HI.

The differential metabolites of the 3 comparison groups were enriched in Kyoto Encyclopedia of Genes and Genomes (KEGG) pathways (**Figure 3**). Hypoxia affected the metabolisms of vitamins (nicotinamide/VB_3_, biotin/VB_7_, thiamine/VB_1_, folate/VB_9_ and VB_6_) and amino acids (histidine, lysine, arginine, phenylalanine, tyrosine, tryptophan, proline, valine, leucine, isoleucine and β-alanine) (**Figure 3A**). Under infection, the metabolism of serotonergic synapse was influenced. Moreover, taurine metabolism involved in bile acid synthesis was also modulated (**Figure 3B**). In addition to some of the above metabolites, sphingolipid was found to be regulated under hypoxia and infection (**Figure 3C**). These results indicate that the metabolisms of vitamins and amino acids are important for the host to resist hypoxia and infection.

### Characterization of Differential Proteins

**Figure 4:**
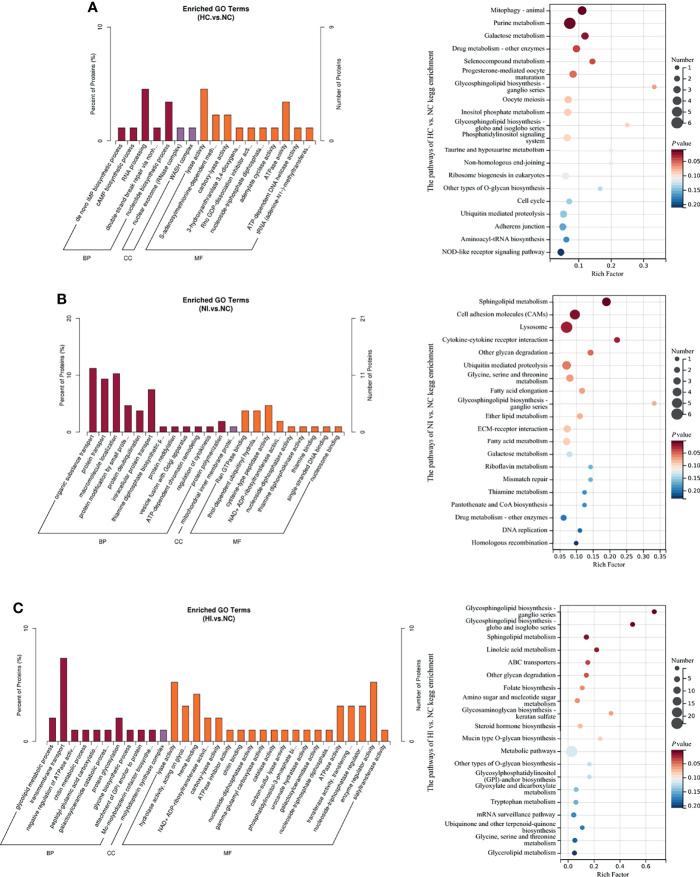
The enriched GO terms (*P* value < 0.05) and top 20 KEGG pathways sorted by *P* value based on the differentially expressed proteins of the three compared groups. Percent of proteins: the ratio of *x* to *n*. The size of the point indicates the number of enriched metabolites. Rich Factor, the ratio of *x* to *M*; BP, biological process; CC, cellular component; MF, molecular function. **(A)** The enriched GO terms and KEGG pathways of NC versus HC. **(B)** The enriched GO terms and KEGG pathways of NC versus NI. **(C)** The enriched GO terms and KEGG pathways of NC versus HI.

The enriched Gene Ontology (GO) terms and KEGG pathways of differential proteins indicated cellular processes under hypoxia or (and) infection of *V*. *parahaemolyticus* (**Figure 4**). Under hypoxia, the main processes focused on RNA processing, lyase activity and ATPase activity, and the activities of many other enzymes were influenced. The enriched pathways focused on mitophagy, purine metabolism, galactose metabolism, drug metabolism-other enzymes and selenocompound metabolism. Interestingly, nucleotide-binding oligomerization domain (NOD)-like receptors (NLRs) signaling pathway was regulated (**Figure 4A**). Under infection, the main processes focused on the modification (deubiquitination, ADP-ribosylation and neddylation) and transport of proteins. The enriched KEGG pathways focused on sphingolipid metabolism, cell adhesion molecules, lysosomes, cytokine-cytokine receptor interactions and glycan degradation. Consistent with the metabolites, thiamine metabolism was influenced. Other vitamins like riboflavin/VB_2_ and pantothenic acid/VB_5_ were also modulated. In addition, fatty acids and galactose were also regulated (**Figure 4B**). Under hypoxia and infection, the main processes of transmembrane transport of substances and the regulation and activity of enzymes were seriously influenced. The enriched KEGG pathways focused on sphingolipid metabolism, glycosphingolipid biosynthesis, linoleic acid metabolism, ABC (ATP-binding cassette) transporters and mucin type *O*-glycan biosynthesis (**Figure 4C**), partially overlapped with the reactions under infection. Apparently, more biotic activities are influenced, which emphasizes the complexity of the effects of double stressors. According to association analysis between metabolome and proteome, five metabolites and 6 proteins were involved in purine metabolism under hypoxia, while 4 metabolites and 2 proteins were involved under hypoxia and infection (**Tables S1–3**). This indicates that purine metabolism is important for resisting infection under hypoxia.

### Theoretical Signaling Pathways in Response to Hypoxia or (and) Infection of *V*. *parahaemolyticus*

**Figure 5:**
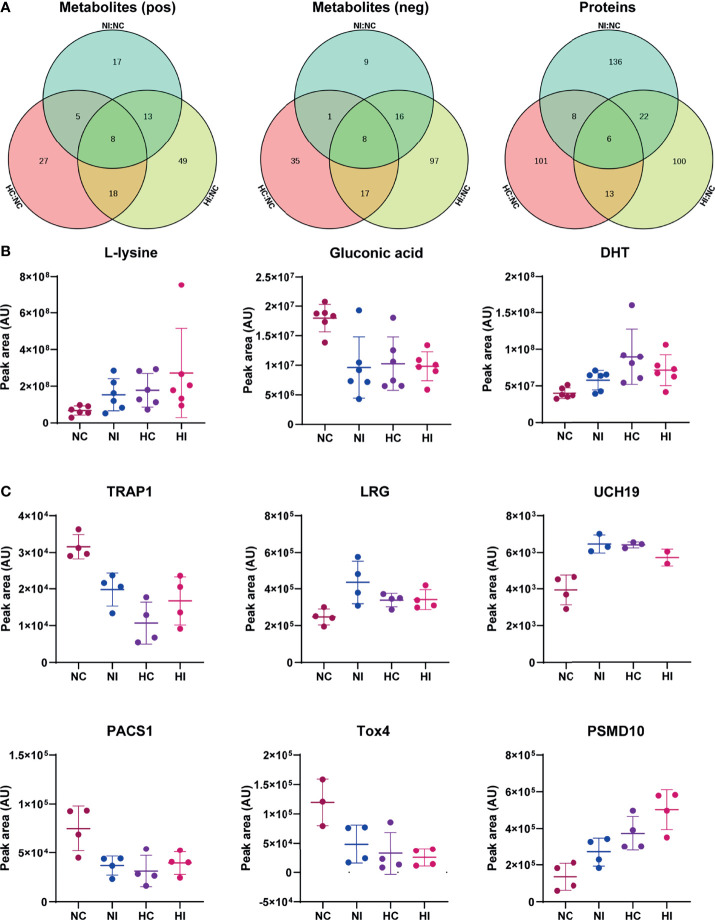
Common differential metabolites and proteins. The peak area was used for relative quantification. **(A)** Venn diagram of differential metabolites of the three compared groups (NC versus NI, NC versus HC, NC versus HI) in positive ion scanning mode; Venn diagram of differential metabolites of the three compared groups in negative ion scanning mode; Venn diagram of differential proteins of the three compared groups. **(B)** Three of the common differential metabolites. DHT: 5α-dihydrotestosterone. **(C)** The common differential proteins. TRAP1, TGF-β receptor-associated protein 1; LRG, leucine-rich alpha-2-glycoprotein; UCH19, ubiquitin C-terminal hydrolase 19; PACS1, phosphofurin acidic cluster sorting protein 1; Tox4, TOX high mobility group box family member 4; PSMD10, 26S protein non-ATPase regulatory subunit 10; AU, arbitrary unit. Error bars on graphs indicate mean ± SD.

**Figure 6:**
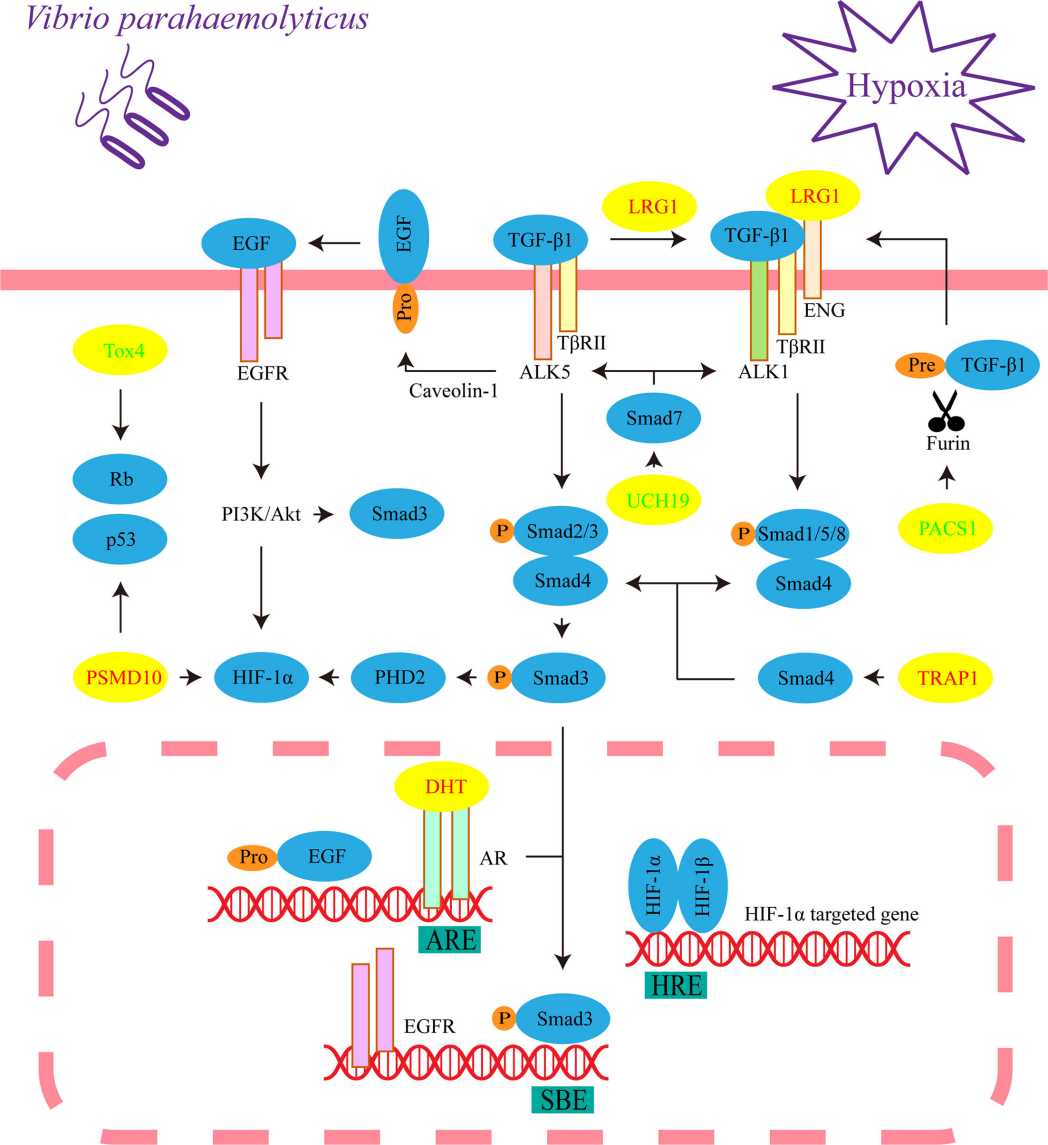
Critical signaling pathways in response to hypoxia and/or *V*. *parahaemolyticus* infection. This represents the cellular process of liver cells under three treatments (hypoxia, infection, and infection under hypoxia). The yellow ellipses indicate differential metabolites or proteins. The increased metabolites or proteins are shown in red font, while the decreased metabolites or proteins are shown in green font. ENG, endoglin.

All identified differential metabolites and proteins were used to draw Venn diagrams, which revealed that there were 22 metabolites and 14 proteins that changed under hypoxia or infection of *V*. *parahaemolyticus*. Sixteen metabolites and 6 proteins also changed under simultaneous stressors (**Figure 5A**). Importantly, the changing trends of these 22 metabolites or proteins were consistent in the three pairs of groups, which indicated that hypoxia and bacterial infection may aggravate each other. We examined the specific functions of these 22 metabolites or proteins and found that seven of them were principally involved in TGF-β1, hypoxia-inducible factor-1α (HIF-1α), and epidermal growth factor (EGF) signaling pathways (**Figures 5B, C**). Finally, a theoretical model of the host in response to hypoxia and bacterial infection was drawn through mechanisms that had been or had not been determined (**Figure 6**).

## Discussion

### Enriched KEGG Pathways and GO Terms

According to the results of KEGG pathways and GO terms, B vitamins were found to play an important role in the effects of hypoxia and infection. VB_1_-derived molecules are important cofactors for numerous enzymes involved in energy production *via* the tricarboxylic acid (TCA) cycle (19). VB_2_ may promote the proliferation of neutrophils and monocytes and activate macrophages to enhance host resistance to infection (20). Sirtuin 1, an inhibitor of non-redox enzymes that helps cells survive under various stress conditions, converts nicotinamide adenine dinucleotide (NAD) to nicotinamide/VB_3_ (21). VB_5_ is necessary for coenzyme A (CoA) synthesis, and CoA is a key cofactor in TCA cycle and fatty acid metabolism (19). VB_6_ can be used as an inhibitor of cell proliferation (22), and VB_7_ deficiency is associated with increased inflammation (23), while VB_9_ deficiency has been shown to inhibit the proliferation of primary human CD8^+^ T lymphocytes (24). Taken together, B vitamins are the key factors that regulate immunity under hypoxia and bacterial infection. In addition, amino acid homeostasis was destroyed. A variety of amino acid metabolism was regulated, and lysine was significantly increased under hypoxia and infection (**Figure 5B**), however, little is known about its specific role.

Pattern recognition receptor NLRs signaling pathway was regulated under hypoxia, indicating that hypoxia may affect the host innate immunity against pathogens. Under infection, the metabolisms of serotonin and bile acid were modulated, and this might have regulated virulence of *V*. *parahaemolyticus* (25, 26). Sphingolipid, an important component of biofilm structure, plays an important role in cell signaling, and *V*. *parahaemolyticus* has been demonstrated to destroy the integrity of the host cell membrane (27). The other cellular processes enriched by KEGG pathways and GO terms may partly be attributed to the effectors secreted by *V*. *parahaemolyticus* because the identified effectors of the type III secretion system (T3SS) have been proven to control the cellular processes of the host (28). Under hypoxia and infection, mucin-type *O*-glycans was regulated, which was considered to have critical roles in defending mucus barrier integrity, and therefore regulated host–microbe interactions at mucosal sites (29). Taken together, these results represented the effects of hypoxia and infection on the host.

### LRG Regulates TGF-β1 Signaling Pathway

We assigned TGF-β receptor-associated protein 1 (TRAP1), LRG, ubiquitin C-terminal hydrolase 19 (UCH19) and phosphofurin acidic cluster sorting protein 1 (PACS1) to TGF-β signal transduction, which is essential to maintain the homeostasis and normal immune function of the liver, pancreas and gastrointestinal system (30). The compound formed by TGF-β receptor I (TβR I, also known as activin receptor-like kinase, ALK) and TGF-β receptor II (TβR II) recognizes TGF-β and then phosphorylates the C-termini of the transcription factors Smad2 and Smad3. The common mediator Smad4 binds to phosphorylated Smad2 and Smad3 and translocated into the nucleus to regulate target gene expression (31). In the above processes, TRAP1, as a molecular chaperone of Smad4, assists it in moving to the compound and binding to Smad2 (32).

LRG1 is a highly conserved member of the leucine-rich repeat family of proteins and promotes the transition of TGF-β1 signaling from Smad2/3 to Smad1/5/8 signaling transduction, which leads to cell proliferation and pathogenic angiogenesis (33). Interestingly, LRG was significantly increased in the liver and significantly decreased in the heart in COVID-19 autopsies (3). Some recent studies indicated that LRG involved in regulations of EGF and HIF-1α signaling pathways (34, 35), and modulated kidney fibrosis (36). Therefore, we propose that LRG is widely involved in the transition of the TGF-β1/Smad signaling pathway in response to hypoxia and pathogenic infection in vertebrates as a conserved molecule.

Eukaryotes utilize the ubiquitin proteasome system (UPS) to degrade proteins, and TGF-β/Smad signal transduction is blocked by inhibitory Smad7, which recruits the ubiquitin ligase Smurf2 to form a complex that degrades the TβR compound through the UPS (37). However, ubiquitination is reversed by the deubiquitinating enzyme UCH37, which competitively binds Smad7 (38). In addition, UCH37 stabilizes Smad2/3 and promotes TGF-β1 signal transduction (39). Similarly, we propose that the reduction of UCH19 may promote the transition of TGF-β from Smad2/3 to Smad1/5/8 signaling transduction. A pathway is found to prevent the excessive activation of TGF-β signal transduction in the model. Furin, a member of the proprotein convertase family, is located in the *trans*-Golgi network (TGN), which catalyzes the maturation of TGF-β1 (40, 41). PACS1 is involved in controlling the correct subcellular localization of furin (42). Therefore, the reduction in PACS1 may inhibit the release of mature TGF-β1 by affecting the correct localization of furin to the TGN.

Fibrosis in the portal area of the liver may be related to the signal transduction of TGF-β1 (**Figure 1C**). It is believed that inflammatory monocytes, tissue-resident macrophages, activation of TGF-β1, cytokines and recognition of the microbiome by pattern recognition receptors all contribute to fibrosis (43). Repairs after injuries are related to the accumulation of collagen and fibronectin in the extracellular matrix. When the injuries are excessive, the accumulation of these components leads to excessive fibrosis (43). The accumulation is closely related to TGF-β1 (44). The activation of TGF-β1 mediated by integrin in the extracellular matrix results in activation of classical Smad signaling and Smad-independent signaling pathways, and they contribute to the expression of profibrotic genes and other transduction of signaling related to fibrosis (45).

### EGF Signaling Pathway

Considering the correlation between protein kinase B (Akt) and Smad3, we assigned 5α-dihydrotestosterone (DHT) to the EGF signaling pathway, which plays an essential role in impairing apoptosis dominated by TGF-β1/Smad3 (46). The proapoptotic activity of the transcription factor Smad3 in hepatocytes requires nuclear translocation governed by C-terminal phosphorylation, and the activation of p38 mitogen-activated protein kinase is also required. In the nucleus, Smad3 enhances apoptosis by binding the promoter of Bcl-2 (an important inhibitor of apoptosis) to inhibit its transcription (47). However, Akt interacts directly with unphosphorylated Smad3 in the cytoplasm. At the same time, Akt phosphorylates and therefore inhibits the activities of some upstream kinases of p38 signaling (48–50). We propose that Akt may hinder the phosphorylation and nuclear translocation of Smad3.

Caveolin-1 (a structural protein of caveolae) and TGF-β1 mediate the correct localization and activation of metalloprotease TACE/ADAM17, which is necessary for the activation of EGF signaling (51, 52). After translocating to the nucleus, the complex formed by DHT and androgen receptors (ARs) may directly bind to the androgen response element (ARE) in the promoter region of the EGF gene, which simultaneously increases the expression of the EGFR gene (53–55). Interestingly, translocated Smad3 binds to the Smad-binding element (SBE) of the EGFR promoter and is rarely responsible for transcriptional activation (56). However, the DHT/AR complex inhibits TGF-β1/Smad3 transcriptional responses by repressing the binding of Smad3 to SBE (57). In conclusion, DHT may prevent the phosphorylation and nuclear translocation of Smad3, therefore inhibiting the apoptotic effect of TGF-β1/Smad3 signaling by activating EGF/phosphatidylinositol-3-kinase (PI3K)/Akt, this seems to be synergistic with LRG.

### Bidirectional Regulation of HIF-1α Signaling Pathway

In this model, TGF-β1 and EGF signaling indicate a response of bidirectional regulation to HIF-1α. Under normoxic conditions, the prolyl hydroxylase PHD2 hydroxylates two proline residues of HIF-1α, and HIF-1α is degraded through the UPS (58, 59). However, hypoxia hinders this process, causing HIF-1α to translocate to the nucleus and form a dimer by interacting with HIF-1β. Then, the dimer binds to the hypoxia response element (HRE) to regulate gene transcription (60). TGF-β1 inhibits the gene transcription of PHD2 by activating Smad2/3 (61); therefore, the transition of TGF-β1 signaling from Smad2/3 to Smad1/5/8 signaling transduction may relieve this inhibition and ultimately promote the degradation of HIF-1α. In contrast, DHT induces PI3K/Akt activation through EGF signaling and promotes HIF-1α gene transcription and protein synthesis (53). In the proteomic results, HIF-1α is not a significantly differential protein, and this supports the bidirectional regulation.

### Cell Proliferation, *V*. *parahaemolyticus* and Cancers

At present, cell proliferation seems to be a significant event under hypoxia and infection. Interestingly, the last two common differential proteins, TOX high mobility group box family member 4 (Tox4) and 26S protein non-ATPase regulatory subunit 10 (PSMD10, also known as gankyrin), reflect an association with Rb and p53. Rb and p53 are two key tumor suppressors that inhibit abnormal cell proliferation, and PSMD10 is their negative regulator (62, 63). Therefore, PSMD10 is overexpressed in numerous types of cancers, including hepatocellular carcinoma, breast cancer and pancreatic cancer (62, 64, 65). PSMD10 has been demonstrated to promote the proliferation, invasion, metastasis and angiogenesis of cancer cells through PI3K/Akt/HIF-1α signaling, which may depend on promoting the degradation of HIF-1α (66, 67). Phosphatase nuclear targeting subunit (PNUTS) is one of the target genes of HIF-1α, and it regulates the phosphorylation of Rb and p53 by binding to protein phosphatase-1 (PP1). Under hypoxia, the separation of PNUTS and PP1 leads to the dephosphorylation and activation of Rb, and the activity of p53 is enhanced (68, 69). Some evidence proposes that Tox4 is capable of binding to PNUTS and PP1 to form complexes; however, it is unclear how Tox4 is involved in regulating the activities of Rb and p53 (70, 71). Another report provides direct evidence that Tox4 inhibits cell proliferation (72). Accordingly, Tox4 may be a positive regulator of Rb and p53. In this model, the decrease in Tox4 and the increase in PSMD10 synergistically promoted cell proliferation. Gluconic acid is associated with cancers. Glucose oxidase reacts with intracellular glucose and O_2_ to produce hydrogen peroxide and gluconic acid (73); therefore, the decrease in gluconic acid means that glucose may be principally utilized for cell proliferation (**Figure 5B**).

Eleven biological agents are clearly designated carcinogens by the International Agency for Research on Cancer. Epstein Barr virus (EBV), Kaposi’s sarcoma herpes virus (KSHV) and *Opisthorchis viverrini* control cell proliferation as one of the carcinogenic mechanisms (74). The reason why *V*. *parahaemolyticus* stimulates the proliferation of host cells partially overlaps with EBV (75). A previous report principally attributed this activity to the effector VgpA of the T3SS of *V*. *parahaemolyticus*. VgpA translocates into host cells and binds to EBV nuclear antigen 1-binding protein 2 (EBP2) in the nucleus (76). c-Myc, the key transcription factors controlling cell growth, metabolism and angiogenesis, form a positive feedback loop to promote cancer cell proliferation with EBP2 (77). We provide evidence to support the purpose of focusing on the role of microbiota in changing the balance between host cell proliferation and death in cancer progression (78), especially those bacteria that deploy additional virulence factors such as *V*. *parahaemolyticus*.

## Conclusion

The main finding of this study is that both of hypoxia and bacterial infection will lead to differences in some metabolites and proteins involved in TGF-β1, EGF and HIF-1α pathways, which may be related to pathological hypoxia caused by inflammation and represent conserved vertebrate signaling pathways. The specific effects of the pathways should be widely studied. In particular, it is necessary to study whether cell proliferation aggravates the metabolic burden. This study provides a basis for understanding how hypoxia destroys the immunity of animals and promotes disease progression.

## Materials and Methods

### Animals and Treatments of Stressors

Five hundred *T*. *obscurus* (length: from 7.5 to 8.0 cm, 10 months old) were purchased from Guangzhou Jinyang Aquaculture Company Limited, China. All fish were allowed to adapt laboratory conditions for 2 weeks. Then 400 *T*. *obscurus* were randomly divided into the NC (100 fish, normal dissolved oxygen concentrations without infection), NI (100 fish, normal dissolved oxygen concentrations with infection), HC (100 fish, hypoxic condition without infection) and HI (100 fish, hypoxic condition with infection) groups. Each group was treated in two repeated water tanks, and each 100 liters of water contained 50 fish. Before formal experiment, the remaining fish were used to determine the survival curve in preliminary experiment to determine the bacterial dose of the formal experiment.

Dissolved oxygen concentrations were controlled by adding nitrogen or O_2_ to the tanks. The dissolved oxygen concentrations of the normoxic and hypoxic groups were 7.60 ± 0.20 mg/L and 2.50 ± 0.20 mg/L, respectively (25.0 ± 0.5°C). *V*. *parahaemolyticus* was grown in Luria-Bertani medium, supplemented with NaCl to a final concentration of 3% (w/v), at 37°C. After reaching the logarithmic growth phase, *V*. *parahaemolyticus* was centrifuged at 5000 x *g* for 5 min and prepared the suspension with phosphate-buffered saline (PBS, Sangon Biotech, cat. no. E607008, China). After 12 h of hypoxic treatment, each fish in the infection group was injected with 5 * 10^7^ cfu *V*. *parahaemolyticus* RIMD 2210633 as described before (the survival curve was measured in the independent preliminary experiment, and 10 *T*. *obscurus* in each infection group were injected with 5 * 10^7^ or 5 * 10^9^ cfu *V*. *parahaemolyticus* according to the same steps) (79, 80), and PBS was used as the control.

### Sample Collection

After infection in the formal experiment, the oxygen concentration was maintained for 12 h. Then, six fish were taken from each group and anesthetized with 200 mg/L tricaine methanesulfonate (BIDE, cat. no. BD234866, China). For metabolomic and proteomic analyses, the entire liver of each fish (2.60 ± 0.32 g) was rapidly cut into small pieces of about 0.5 cm^3^ and transferred to a 15 ml centrifuge tube, then was stored in liquid nitrogen until omics analysis. For pathological observation, another 4 fish from each group were taken and anesthetized, the liver of each fish was rapidly cut into small pieces of about 0.5 cm^3^ and fixed in 4% paraformaldehyde overnight at 4°C before dehydration.

### Pathological Observation

The livers from four fish in each group were treated with Advanced Smart Processor Vacuum Tissue Processor ASP300 S (Leica, Germany) before paraffin embedding. The clearing reagent was turpentine oil. The tissue was then embedded in paraffin and sliced to a thickness of 5 μm using the microtome RM2235 (Leica, Germany). After rehydration, the sections were stained with hematoxylin and eosin (81). And then the sections were mounted and visualized with versatile stereo microscope SMZ 800N (Nikon, Japan).

### Metabolomic Analysis Based on Liquid Chromatography Coupled to Mass Spectrometry (LC–MS)

Considering the heterogeneity of liver tissue, the entire liver was pulverized in a mortar containing liquid nitrogen using a cryogenically cooled pestle for preparing homogenate (liver powder), and 100 mg of homogenate was resuspended in 500 μL of prechilled aqueous solution (80% methanol, 0.1% formic acid, Thermo Fisher Chemical, USA) by vortexing (82). After incubation on ice for 5 min, the mixture was centrifuged at 15,000 x *g* and 4°C for 20 min. Some of the supernatant was diluted to a final concentration containing 53% methanol by adding LC–MS grade water (Merck, cat. no. 1153331000). The samples were then transferred to a fresh centrifuge tube and centrifuged at 15,000 x *g* and 4°C for 20 min. The untargeted LC–MS system included a Vanquish UHPLC system (Thermo Fisher Scientific, USA) and an Orbitrap Q Exactive™ HF-X mass spectrometer (Thermo Fisher Scientific). Samples were injected into a C18 column (Hypesil Gold, 100 * 2.1 mm, 1.9 μm) using a 17-min linear gradient at a flow rate of 0.2 mL/min. Eluent A (0.1% formic acid in water) and eluent B (methanol) were used for positive polarity mode, while eluent A (5 mM ammonium acetate/Thermo Fisher Chemical, pH 9.0) and eluent B (methanol) were used for negative polarity mode. A gradient run was set up as 0-1.5 min at 2% B, 1.5-12.0 min from 2% to 100% B, 12.0-14.0 min at 100% B, 14.0-14.1 min from 100% to 2% B, 14.1-17 min at 2% B. The mass spectrometer was operated with a spray voltage of 3.2 kV, a capillary temperature of 320°C, a sheath gas flow rate of 40 arb and an aux gas flow rate of 10 arb (82).

### Proteomic Analysis Based on Data-Independent Acquisition (DIA) Mode

The homogenate was lysed with PASP lysis buffer (100 mM NH_4_HCO_3_/Merck, cat. no. 5330050050, 8 M urea, pH 8) and ultrasonicated on ice for 5 min. After centrifugation, the supernatant was reduced with 10 mM dithiothreitol (Merck, cat. no. D9163) for 1 h at 56°C. The product was alkylated with moderate iodoacetamide (Merck, cat. no. I6125) for 1 h and then mixed with a fourfold volume of precooled acetone (Beijing Chemical Works, cat. no. 11241203810051, China) at -20°C for 2 h. After centrifugation, the precipitate was obtained and washed with 1 mL of precooled acetone. The final precipitate was collected and dissolved in dissolution buffer (8 M urea, 100 mM TEAB/Merck-cat. no. T7408, pH 8.5). For trypsin treatment, each protein sample was mixed with DB lysis buffer (8 M urea, 100 mM TEAB/Merck, cat. no. T7408, pH 8.5), and the volume was made up to 100 μL, while trypsin (Promega, cat. no. V5280) and 100 mM TEAB buffer were added for digestion (37°C, 4 h). Then, trypsin and CaCl_2_ were added for digestion overnight. Samples were acidified by formic acid and desalted in a C18 column. Peptides were resuspended in loading buffer containing 70% acetonitrile (Thermo Fisher Chemical, cat. no. W6-4) and 0.1% acetic acid. Finally, the eluents of each sample were collected and lyophilized.

Mobile phases A (2% acetonitrile, pH 10.0) and B (98% acetonitrile, pH 10.0) were used to develop a gradient elution. The lyophilized powder was dissolved in solution A and centrifuged at 12,000 x *g* for 10 min at room temperature. The sample was fractionated using a C18 column (Waters BEH C18, 4.6 * 250 mm, 5 μm) on an L3000 HPLC system (RIGOL, China), and the column oven was set as 45°C. The eluates were monitored at UV 214 nm, collected in one tube per minute and finally combined into four fractions. All fractions were dried under vacuum and then reconstituted in 0.1% (v/v) formic acid in water.

For gradient elution, mobile phases A (0.1% formic acid in H_2_O) and B (0.1% formic acid in 80% acetonitrile) were used. A half mixture containing 4 μg of fraction supernatant and 0.8 μL of iRT reagent (Biognosys, Switzerland) was injected into the EASY-nLC 1200 UHPLC system (Thermo Fisher Scientific), and peptides were separated using a 100-min linear gradient at a flow rate of 600 nL/min. MS data were acquired on a Q Exactive™ HF-X mass spectrometer (Thermo Fisher Scientific) operating with a spray voltage of 2.1 kV (Nanospray Flex™ electron spray ionization) and a capillary temperature of 320°C. For data-dependent acquisition (DDA) mode, the m/z range covered 350 to 1500 with a resolution of 120,000 (at m/z 200). The automatic gain control target value was 3 * 10^6^, and the maximum ion injection time was 80 ms. The top 40 precursors of the highest abundance in the full scan were selected and fragmented by higher energy collisional dissociation and analyzed by MS/MS, where the resolution was 15,000 (at m/z 200). The automatic gain control target value was 5 * 10^4^. The maximum ion injection time was 45 ms with a normalized collision energy of 27%, an intensity threshold of 1.1 * 10^4^, and a dynamic exclusion parameter of 20 s. The raw MS detection data were used to construct a DDA spectrum library. For DIA mode, the m/z range covered from 350 to 1500. MS1 resolution was set to 60,000 (at 200 m/z). The full scan AGC target value was 5 * 10^5^, and the maximum ion injection time was 20 ms. Peptides were fragmented by high-energy collision dissociation in MS2, in which the resolution was set to 30,000 (at 200 m/z). The AGC target value was 1 * 10^6^, with a normalized collision energy of 27%.

### Data Processing and Analysis

The raw metabolite data were processed using Compound Discoverer 3.1 (Thermo Fisher Scientific) to perform peak alignment, peak picking, and quantitation. The peaks were matched with the mzCloud (https://www.mzcloud.org/), mzVault and MassList databases. The metabolites were annotated using the KEGG PATHWAY Database (https://www.genome.jp/kegg/pathway.htmL), the Human Metabolome Database (https://hmdb.ca/metabolites) and the LIPID MAPS^®^ Database (http://www.lipidmaps.org/). PCA and PLS-DA were performed at metaX (83). The screening criteria for differential metabolites were VIP > 1.0, fold change (FC) > 1.2 or FC < 0.833 and *P* value < 0.05 (Student’s *t* test). The raw data from the DDA mode were analyzed using Proteome Discoverer 2.2 (Thermo Fisher Scientific) on the basis of the genome of *T*. *rubripes* (https://www.ncbi.nlm.nih.gov/genome/63) to construct the DDA spectrum library, and the raw data from the DIA mode were imported to the DDA spectrum library to obtain chromatographic peaks. The protein quantitation results obtained from the calculation of peak area were statistically analyzed by Student’s *t* test. The screening criteria for differential proteins were FC > 1.2 or FC < 0.833 and *P* value < 0.05 (Student’s *t* test). GO analysis was conducted using InterProScan (European Bioinformatics Institute). KEGG pathway analysis was performed as described above. The specific information on all differential metabolites and proteins can be found in supplementary materials (**Tables S4–12**). Visualizations of Venn diagrams were performed using the OmicShare tools (https://www.omicshare.com/tools). The *P* values of KEGG and GO were obtained by hypergeometric testing, and the formula is as follows:


$$P\;value=1-\sum_{j=0}^{x-1}\frac{\left({}_{j}^{M}\right)\left({}_{n-j}^{N-M}\right)}{\left({}_{n}^{N}\right)}$$

*N*: The number of proteins with GO annotation information in all proteins. *n*: the number of differential proteins in *N*. *M*: the number of proteins annotated to a GO entry in all proteins. *x*: The number of differential proteins annotated to a GO entry. The hypergeometric test for KEGG followed the same principle.

## Data Availability Statement

The datasets presented in this study can be found in online repositories. The names of the repository/repositories and accession number(s) can be found below: http://www.proteomexchange.org/, PXD029027, https://db.cngb.org/, METM0000025.

## Ethics Statement

The animal study was reviewed and approved by Institutional Animal Care and Use Committee (IACUC), Sun Yat-Sen University (Approval No. SYSU-IACUC-2021-B1920).

## Author Contributions

DL and JX conceived the study. JX, HY, XY, SG, ND, and YL carried out the experiment. JX performed the data analysis and wrote the first draft of the manuscript. HL, YZ, and DL acquired funding, and all authors contributed substantially to revisions and approved the final manuscript.

## Funding

This work was supported by the National Key R&D Program of China (2018YFD0900301), Research and Development Projects in Key Areas of Guangdong Province (2021B0202070002), the Science and Technology Planning Project of Guangzhou, China (201904020043), the Guangdong Provincial Special Fund for Modern Agriculture Industry Technology Innovation Teams (2019KJ143), and an Innovation Group Project of the Southern Marine Science and Engineering Guangdong Laboratory (Zhuhai) (311021006).

## Conflict of Interest

The authors declare that the research was conducted in the absence of any commercial or financial relationships that could be construed as a potential conflict of interest.

## Publisher’s Note

All claims expressed in this article are solely those of the authors and do not necessarily represent those of their affiliated organizations, or those of the publisher, the editors and the reviewers. Any product that may be evaluated in this article, or claim that may be made by its manufacturer, is not guaranteed or endorsed by the publisher.
