# Supplementary material for: Comparative Metabolomics and Proteomics Reveal Vibrio parahaemolyticus Targets Hypoxia-Related Signaling Pathways of Takifugu obscurus
Source: Front Immunol. 2022 Jan 13;12:825358. doi: 10.3389/fimmu.2021.825358 (PMC8793131; doi:10.3389/fimmu.2021.825358)
Supplement: Supplementary file 1 [file DataSheet_1.pdf]

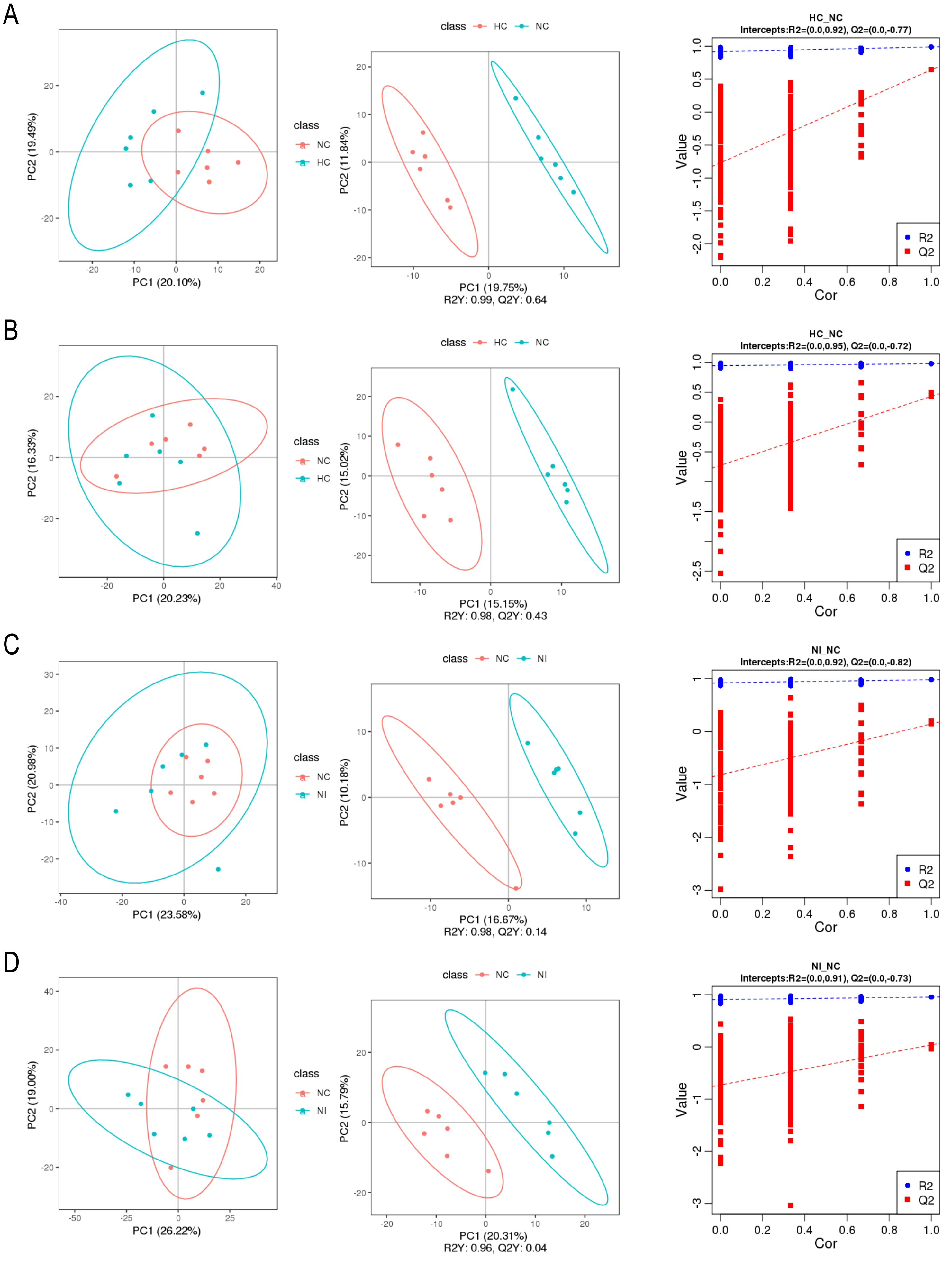
A
PC1 (20.10%)
PC2 (19.49%)
class
NC
HC
class HC NC
PC1 (19.75%)
PC2 (11.84%)
R2Y: 0.99, Q2Y: 0.64
HC_NC
Intercepts:R2=(0.0,0.92), Q2=(0.0,-0.77)
Value
Cor
R2
Q2
B
PC1 (20.23%)
PC2 (16.33%)
class
NC
HC
class HC NC
PC1 (15.15%)
PC2 (15.02%)
R2Y: 0.98, Q2Y: 0.43
HC_NC
Intercepts:R2=(0.0,0.95), Q2=(0.0,-0.72)
Value
Cor
R2
Q2
C
PC1 (23.58%)
PC2 (20.98%)
class
NC
NI
class NC NI
PC1 (16.67%)
PC2 (10.18%)
R2Y: 0.98, Q2Y: 0.14
NI_NC
Intercepts:R2=(0.0,0.92), Q2=(0.0,-0.82)
Value
Cor
R2
Q2
D
PC1 (26.22%)
PC2 (19.00%)
class
NC
NI
class NC NI
PC1 (20.31%)
PC2 (15.79%)
R2Y: 0.96, Q2Y: 0.04
NI_NC
Intercepts:R2=(0.0,0.91), Q2=(0.0,-0.73)
Value
Cor
R2
Q2

Figure S1 The screening of differential metabolites of the NC, HC and HI groups was carried out for quality control and comparisons of differential metabolic characteristics. PC1 and PC2 represent the scores of the first and second principal components, respectively. (A) PCA, PLS-DA and function based on the sevenfold cross-validation method of the NC and HC groups in negative ion scanning mode. (B) PCA, PLS-DA and function based on the sevenfold cross-validation method of the NC and HC groups in positive ion scanning mode. (C) PCA, PLS-DA and function based on the sevenfold cross-validation method of the NC and NI groups in negative ion scanning mode. (D) PCA, PLS-DA and function based on the sevenfold cross-validation method of the NC and NI groups in positive ion scanning mode.
